# Food Neophobia in Children: A Case Study in Federal District/Brazil

**DOI:** 10.3390/nu16172962

**Published:** 2024-09-03

**Authors:** Priscila Claudino De Almeida, Eduardo Yoshio Nakano, Ivana Aragão Lira Vasconcelos, Renata Puppin Zandonadi, António Raposo, Ariana Saraiva, Hmidan A. Alturki, Raquel Braz Assunção Botelho

**Affiliations:** 1 Graduate Program in Human Nutrition, University of Brasília, Brasília 70910-900, Brazil; 2 Department of Statistics, University of Brasília, Brasília 70910-900, Brazil; eynakano@gmail.com; 3 Department of Nutrition, University of Brasília, Brasília 70910-900, Brazil; ivanaunb@gmail.com (I.A.L.V.); renatapz@unb.br (R.P.Z.); 4 CBIOS (Research Center for Biosciences and Health Technologies), Universidade Lusófona de Humanidades e Tecnologias, Campo Grande 376, 1749-024 Lisboa, Portugal; 5 Department of Animal Pathology and Production, Bromatology and Food Technology, Faculty of Veterinary, Universidad de Las Palmas de Gran Canaria, Trasmontaña s/n, 35413 Arucas, Spain; ariana_23@outlook.pt; 6 King Abdulaziz City for Science & Technology, Wellness and Preventive Medicine Institute—Health Sector, Riyadh 11442, Saudi Arabia; halturki@kacst.edu.sa

**Keywords:** child, food neophobia, prevalence

## Abstract

A reluctance to eat and/or avoidance of novel foods is characterized as food neophobia (FN). FN restricts the diet to familiar foods when, in fact, it should be much more varied. FN can be a barrier to healthy foods, affecting the quality of diet, and impairing children’s growth and development. Therefore, according to their caregivers’ perceptions, this study aimed to evaluate FN in children from Federal District/Brazil. The Brazilian Children’s Food Neophobia Questionnaire (BCFNeo), a specific instrument developed and validated in Brazil, was answered by caregivers of children aged 4 to 11 y/o. Sampling occurred through snowball recruitment, being convenient and non-probabilistic. The Health Sciences Ethics Committee approved the study. The analysis evaluated FN in total (BCFNeoTot) and in the following domains: general (FNgen), for fruits (FNfru), and for vegetables (FNveg). FN scores were compared between sex and child’s age and categorized according to three ordinal levels. FN levels were compared using the Mann–Whitney U test. The Friedman test, followed by the Wilcoxon test with Bonferroni correction, was performed to analyze differences in FN according to the environment. Of the caregivers’ answers for their children, 595 answers were included, because 19 were out of age. The prevalence of high FN was 42.9%. The domain with the highest prevalence of high FN was vegetables (48.6%). Children aged 8 to 11 y/o had a higher mean FN in two domains (FNgen *p* = 0.047 and FNveg *p* = 0.038) when compared to children aged 4 to 7 y/o. Boys were more neophobic in all domains (FNgen *p* = 0.017; FNfru *p* = 0.010; FNveg *p* = 0.013; BCFNeoTot *p* = 0.008), and FN tends not to decrease with age. The results showed that the children of the FD are more neophobic than Brazilian children in general, highlighting the importance of additional studies in FN determinants in this population and nutritional education interventions to reduce FN among FD children.

## 1. Introduction

A reluctance to eat and/or avoidance of novel foods is characterized as food neophobia (FN) [1]. This condition can restrict the diet to familiar foods when, in fact, it should be much more varied, especially for omnivores like humans. Therefore, FN describes a psychological trait whose intensity varies among individuals, raising questions of causality, correlation, and measurement [2].

Despite FN being a relevant topic in health promotion, it began gaining prominence in the late 20th century, particularly during the 1980s and 1990s. The first instrument to evaluate FN was created in 1992 by Pliner and Hobden [1]. In 1994, Pliner created an instrument focused on children [3]. It is relevant to choose the specific instrument, target group, and ways to measure this behavior [4].

FN intensely affects the consumption of healthy food, and it is a major barrier to consuming fruits and vegetables [5], affecting the diet quality and promoting deficiencies of certain essential nutrients, especially minerals and vitamins [6,7]. Children with FN may not acquire some of the skills associated with eating [6]. Deficiency or excess of some nutrients and malnutrition are critical and can lead to irreversible health consequences [6]. Children with neophobic behavior, who consume fewer and less-varied fruits and vegetables, are at a higher risk of becoming overweight [8]. Once FN is observed and quantified, strategies can be developed to overcome it, and valuable changes can occur in childhood and have significant impacts on adult life, such as obesity prevention [9].

Compared to other children from several countries, neophobic children were less likely to eat raw or cooked vegetables and legumes [10], berries [11], grapes [12], eggs [10], chicken [12], fish [11], cheese [12], and proteins [13]. They tend to eat more sweets [10], snacks [10], and fast food [14]. The energy consumption of neophobic children can be lower [12] or higher [10] or does not have significant differences among food neophobia levels, with a high intake of carbohydrates [10], lower intake of cholesterol [13], vitamin C [10], potassium [13], thiamin [10], phosphorus [13], folate [10], magnesium [13], iron [13], zinc [13], and selenium [13].

Some authors state that the distribution of the percentage of carbohydrates (50%) and fats (33%) does not significantly differ among the different levels of neophobia. However, the percentage of protein consumed by children is lower with increasing levels of neophobia (low: 16%; medium: 15%; high: 14%), possibly associated with higher sensory sensitivity towards protein-rich foods [13]. In addition, a higher degree of food neophobia also was not associated with lower consumption of functional foods (which included foods rich in bioactive compounds such as antioxidants, dietary fiber, omega-3 fatty acids, plant sterols, prebiotics, and probiotics) nor with a higher perceived risk associated with these foods [15]. Food neophobia is complex [4] and has many associated factors [16], such as gene traits [5] and prenatal experiences [17], flavor exposure in utero [5], flavor exposure during breastfeeding [5], parental influence on children’s eating habits [16], heredity [5,18], sensory preferences [17], cognitive schemata [5], children’s innate preference for sweet and savory flavors, the influence of the sensory aspect of the food, parents’ pressure for the child to eat, parents’ lack of encouragement and/or affection at mealtime, childhood anxiety, and feelings and emotions [16].

FN is common in childhood worldwide, especially at moderate and high levels [16]. This behavior is related to severe dietary restrictions and impacts on health [16], and it is expected between 2 and 3 years old [17] and between 2 and 5 years old [16]. After this period, FN tends to decrease until stabilization in adulthood [16,17]. In Brazil, previous studies showed that FN did not decrease with age among children from 4 to 11 years old [19], and the dietary intake of Brazilian children (0 to 10 y/o) had a high prevalence of inadequate micronutrient intake and poor diet quality [20]. According to the Brazilian Institute of Geography and Statistics (IBGE) [21], children consume fruits, vegetables, pork, roots, and tubers less frequently and consume more cookies, chips, soft drinks, ham, and sausages [21]. These worrying situations highlight the need for more research in the country.

Despite the studies performed in Brazil [19,22], no specific studies about FN in children in the Federal District (FD)/Brazil were observed [19,22]. The FD is located in a rich savanna with lots of biodiversity, being part of the second largest biome in South America, named “cerrado” [23]. Of the total residents of the FD, 55.5% were born locally, and nearly half in other Brazilian states [23]. Biodiversity and cultural diversity, represented by people from other Brazilian states, can be seen in the variety of local foods, which increases the chances for children to be exposed to different foods.

Thus, our study hypothesizes a high prevalence of food neophobia, which decreases with age, in both girls and boys living in the Federal District. Therefore, according to their caregivers’ perceptions, the study aimed to assess food neophobia in children residing in the Federal District of Brazil. Considering the consequences and risks inherent to FN, those involved in multiprofessional follow-up and all caregivers involved with children need to be aware of it [16], and specific knowledge about this FN may allow more effective interventions [22].

## 2. Materials and Methods

It is a descriptive cross-sectional study. It was conducted with children (4 to 11 years old) from the FD. Following the Declaration of Helsinki guidelines, the study was analyzed and approved by the Health Sciences Ethics Committee, University of Brasilia, No. 5.438.498.

### 2.1. Participants

Sampling occurred through snowball recruitment, being convenient and non-probabilistic. The study included caregivers of Brazilian children (4 to 11 y/o) who live in the FD/Brazil. They needed to agree to participate and know about the child’s eating behavior. The consent form had the conditions for participation in the research. Researchers excluded incomplete questionnaires.

Since there are no specific data on the FD population from 4 to 11 y/o, the minimum sample size was calculated considering 10 individuals per instrument item [24], resulting in 250 participants. Considering a significance level of 5% and power of 80%, this sample size guarantees that an effect size of 0.2 (small/medium) for the difference between the two groups will be statistically significant.

### 2.2. Instruments and Application

The Brazilian Children’s Food Neophobia Questionnaire (BCFNeo) was chosen as it is still the only validated instrument in Portuguese to be used with caregivers of Brazilian children aged 4 to 11 years [8,9]. The instrument was composed of sociodemographic data, and BCFNeo [9] was available through the online platform Google forms^®^. We used social networks (such as Instagram^®^, Facebook^®^, and Twitter^®^), messaging apps, and email to reach participants. The data collection process occurred from November 2020 to November 2022.

To begin, the caregivers were questioned about their sociodemographic aspects (sex, age, degree of kinship, marital status, and educational level) and then about their children’s (sex, nationality, diagnoses, age, and family income). Caregivers answered the instrument on their own. The instruction that only one caregiver should answer the instrument was given, and the caregiver could answer more than once if there was more than one child in the age group. The financial values, when presented, considered the conversion rate of USD 1.00 to BRL 4.93, the conversion value in January 2024. Considering the same conversion rate, the minimum wage (MW) for the analysis was BRL 1412.00, about USD 285.

The categorization of FN score followed a protocol previously validated [6]. The items were divided into three domains: neophobia in general, for fruits, and for vegetables. The domains had a similar number of items. Therefore, a better analysis of the score of the entire instrument (25 items) and each domain was performed. Each domain separately or the complete instrument allowed the assessment of the neophobic traits [6].

Data obtained from each item assumed values from 0 to 4. The general domain score comprises 9 items and presents punctuation from 0 to 36. The fruit FN score comprises 8 items with punctuation varying from 0 to 32. The FN vegetable domain score consists of 8 items and ranges from 0 to 32. Considering all domains, the FN total score comprises 25 items, and the total punctuation varies from 0 to 100 (9). The categorization of FN was defined according to scores: (i) up to 40 points: low neophobia, (ii) from 41 to 65 points: moderate neophobia, and (iii) 66 points or more: high neophobia. Therefore, for each domain, a low FN is up to 13 points, a moderate FN is between 14 and 21 points, and a high FN is from 22 points [6].

For some analyses, specific items of the instrument were used. These items refer to three environments children are exposed to: school, home, and a friend’s home, influencing FN.

### 2.3. Statistical Analysis

The analysis used two age groups (4–7 and 8–11) and sex (female and male). The neophobia scores were compared between the sex and ages of the children (Student’s *t*-test). These scores were also categorized according to three ordinal levels, and the Mann–Whitney U test was used to compare neophobia levels between sex and child’s age. The Friedman test, followed by Wilcoxon test with Bonferroni correction, was performed to analyze differences in FN according to the environment by sex and child’s age. The normality of the observations was tested using the Kolmogorov–Smirnov test with Lilliefors correction. All tests considered bicaudal hypotheses and a significance level of 5%. After extraction from the Google Forms^®^ platform, analysis was performed using the SPSS^®^ 20.0 software and Excel^®^ 16.67. Descriptive statistics were presented as mean and standard deviation or frequencies and percentages.

## 3. Results

The sample comprised 595 participants after excluding 19 responses outside the age range (96.9% of individuals who accessed and completed the study were included). Caregivers were primarily female (n = 558; 93.8%); married or in a stable relationship (n = 489; 82.2%); living in urban areas (n = 586; 98.5%), aged between 19 and 66 y/o (mean age 39.41 ± 7.03 y/o). Most caregivers (Supplementary Table S1) were mothers (n = 534; 89.7%) with postgraduation (n = 255; 42.9%). The mean number of people living in the same house was 3.86 ± 0.97. Most (n = 328; 55.13%) had a monthly family income of more than ten minimum wages.

Regarding sex and age (Table 1), the sample is well balanced. Most children were boys (n = 336; 56.5%); the mean age was 6.93 ± 2.29 y/o. Most children (n = 367; 61.68%) had no medically diagnosed conditions; 228 (38.31%) presented one or more medical diagnoses.

The prevalence of low and high FN was 24.5% and 42.9%, respectively (Table 2). The domain of neophobia for fruits was the one with the lowest prevalence of high neophobia (37.0%), and the domain for vegetables had a higher neophobia prevalence (48.6%).

Table 3 shows that the mean score for FN was higher for children aged 8 to 11 y/o in the general domain (*p* = 0.0047) and in the vegetable domain (*p* = 0.038). High neophobia was most prevalent for boys in the general domain (*p* = 0.011), in the fruit domain (*p* = 0.005), in the vegetable domain (*p* = 0.001), and in total (*p* = 0.023).

To analyze if the environment (school, home, or a friend’s house) could influence FN, we used specific items from the instrument that assessed FN for fruits and vegetables and based on the friend’s influence, for these three environments. Each item has a score from 0 to 4 points.

Table 4 shows that children, in general, had lower FN towards fruits at school (*p* = 0.000), higher FN towards vegetables at a friend’s house (*p* = 0.000), and, when under the influence of a friend, there was no difference (*p* = 0.248) between environments (home, school, and a friend’s house). Younger children (4–7 y) had lower FN towards fruits (*p* = 0.000), vegetables (*p* = 0.000), and with friend´s influence (0.005) at school. Children ≥8 y/o had lower FN with friend´s influence at home (*p* = 0.031). Girls and boys had lower FN towards fruits at school (*p* = 0.008; *p* = 0.005), and boys had higher FN towards vegetables at friend´s houses (*p* = 0.001).

Evaluating year-by-year and sex differences (Supplementary Table S2), total FN scores ranged from 52.42 to 65.14. Except for five and eight y/o, male children presented higher total FN. This was also observed in the FN of fruits and vegetables.

## 4. Discussion

This study evaluated FN in children from the Federal District/Brazil, following the perception of their caregivers. The prevalence of high FN was higher (42.9%) than in Brazil (33.4%) [19]. The analysis by age groups showed that older children (8–11 y/o) were more neophobic than younger (4–7 y/o). The difference was significant (*p* < 0.05) in vegetables and general neophobia. These results have not been confirmed in other local Brazilian studies [25,26]. In the first Brazilian study, evaluating children from 3 to 13 years in Uberaba/Minas Gerais, the youngest children tended to have the lowest interest in food, such as slow ingestion and satiety response. However, the authors found no significant difference in FN in the age group [25]. The second Brazilian study, performed with children aged 3 to 6 years in Natal/Rio Grande do Norte, found a greater tendency to FN in children aged 3–4 years compared to those aged 5–6 years (*p* < 0.000) [26]. A national study in Brazil found no significant difference in FN with age [19].

On the other hand, research in other countries has shown a reduction in FN as age advances. A study evaluated 5 national surveys conducted in Ireland on 3246 people aged 1 to 87 years and found that FN increased with age from 1 to 6 years, then decreased until early adulthood, where it remained stable until increasing with age in older adults (>54 years) [27]. An integrative review with a systematic approach showed a lack of patterns regarding sex or age with higher degrees of FN in children [8]. In our study, boys are more neophobic than girls, with a higher mean in general neophobia (*p* = 0.017), fruit neophobia (*p* = 0.010), vegetable neophobia (*p* = 0.013), and total FN (*p* = 0.008).

In Poland, 325 children from 2.5 to 7 years old [10], and in the United States, 85 children aged 3 to 12 years [28] were assessed, and no sex-related differences were found for FN. Unlike this study, two population studies conducted in Brazil and another in Ireland showed that FN was more prevalent among boys than girls [19,27]. Brazilian local studies varied in this regard. One of them did not find significant differences for FN concerning sex [25], and the other mentioned that girls aged 3–6 years were more neophobic than boys (*p* = 0.0063) [26].

The results on neophobic behavior and sex differences are difficult to explain. The studies that showed sex differences diversified on the justification [19,25,26]. When boys were more neophobic, the explanation was related to food preferences, suggesting that boys prefer less healthy foods, such as more meat and fatty foods, and girls prefer more fruits and vegetables. Furthermore, the sex variable can be determined by environmental effects such as personality [19].

When the girls were more neophobic, the explanation was related to the protective effect of neophobia and the role of women historically in choosing, organizing, and preparing the food offered to the families. Therefore, being more predisposed to avoid the new, they are more cautious, and this is a selective incentive that stimulates the most neophobic response [26]. A study suggested that FN was attributable to genetic factors, explaining approximately two-thirds of the variation in females [29].

Studies evaluated dietary intake in neophobic individuals and showed that energy intake among the levels of neophobia can be higher [10], lower [12], or equal [7]; that the percentage of carbohydrates can be higher (8) or equal [7]; and that dietary fat (including essential fatty acids) can be equal [7], but protein intake may be lower in more severe cases of neophobia [7]. Additionally, the low intake of fruits and vegetables may lead to a reduced intake of micronutrients such as vitamin C [10], potassium [13], thiamin [10], phosphorus [13], folate [10], magnesium [13], iron [13], zinc [13], and selenium [13]. However, neophobic individuals tend to eat more sweets [10], snacks [10], and fast food [14]. Some studies evaluated neophobia levels and did not find an association with lower consumption of functional foods [8], but the adherence to the Mediterranean diet—characterized by “high consumption of fresh or dried fruits and vegetables, legumes, and whole grains; moderate consumption of fish, dairy, and meat; low-to-moderate intake of red wine during meals; and the use of olive oil as the primary source of fat”—was inversely associated with food neophobia, suggesting that this personality trait may affect dietary pattern [13].

In Brazil, the federal units are different in geography, climate, food production, culture, and habits due to Brazilian territorial extension. The development of agriculture in the FD enhanced food production, and food became more accessible in terms of price and proximity to purchasing fresher food [30,31]. Greater access to a large variety of food does not necessarily mean the diet will be healthier. Most of the children in this study are from high-income families. The analysis of Brazilian food consumption [21] in children over ten y/o showed that the frequency of food consumption is more prominent in the higher-income strata. However, a higher per capita consumption of negative markers of diet quality (i.e., consumption of sweets, pizzas, fried and baked snacks, sandwiches) was observed in people with higher incomes, and a lower frequency of consumption of foods such as rice, corn, corn-based preparations, beans, pasta, and poultry [21].

In Brazil, four y/o children must be enrolled in early education and, at six, in elementary school [32,33]. There are studies [17,34] that considered social and environmental factors. These might also significantly influence and modulate children’s food avoidances because the child may or may not feel safe in surroundings with unfamiliar food. Furthermore, there is growth in the potential of performing a specific behavior that a peer performs, or the teacher or parents may encourage to perform. For this reason, our study analyzed the influence of the different living surroundings (school, home, and friends’ house) on FN of fruits and vegetables and friends’ consumption of new foods. Our results showed that school may be the best place to stimulate the consumption of new fruits and vegetables for younger children and new fruits for girls and boys [17,34].

This result differed from the study by Damsbo-Svendsen et al. [34], which indicated that most children responded indifferently to unfamiliar foods at school (39.6%) or the youth center (30%). On the contrary, most children answered that they would surely try these foods at home (47.2%), at a relative’s house (46.4%), and a friend’s house (44.7%) [34]. Our study was carried out according to the caregivers’ perception, and the above-mentioned study was carried out directly with the children.

In our study, the low FN prevalence of 24.5% was less than observed in Brazilian children (39.9%) [19]. The prevalence of high FN in FD was higher (42.9%) than in Brazil (33.4%) [19], indicating that children from FD could be more neophobic, and future research is necessary to evaluate the causes, allowing to change this scenario with one-off strategies and policies. Higher levels of FN, when persistent, should be included in the clinical domain as a subtype of eating disorders, leading to disruptions to personal and social life [5].

This study presents limitations to the Snowball Sampling (SS) method used to spread the research. SS is a cheap and popular sampling method in social research [35] that can be quickly disseminated. However, a strength of this method is that a minimum sample was defined before data collection to be representative. The sample may have reflected the SS method with most postgraduate caregivers and a high family income. However, these results can be explained since the FD was the federal unit with the highest human development index (0.814) and the country’s highest monthly per capita income in 2022, about USD 680 (BRL 3357.00) [36].

Despite the limitation of convenience sampling [37] in this local study, in one city, we included children with food allergies and intolerances (representing 11.26% of the sample) and other conditions, as well as prevalence data for other studies in the FD. Other studies on FN usually do not bring data relating FN and food allergies or intolerances [11,19].

Another potential study limitation relates to the scale used to measure food neophobia. It is an indirect approach in which the questions pertain to what the caregiver thinks the child’s behavior would be in a given scenario, rather than presenting the situation and directly assessing the child’s reaction [2]. Caregivers’ perceptions depend on memory, but it is believed that these caregivers, primarily mothers, often have extensive and usually quite comprehensive exposure to the eating habits, likes, and dislikes of the individuals under their care. Another possible research method for the study’s subject would be similar to the one used in the prospective study by Nicklaus et al., 2005 [38], which involved behavioral measures through observing French children’s lunches. However, this type of study also has limitations and is difficult to operationalize, which would significantly limit the studied sample size [2,38]. Additionally, Alley (2018) [2] highlights that the scores of scales used to assess food neophobia seem to possess strong psychometric properties, including predictive validity [2].

Food neophobia (FN) is a significant issue for children who suffer from conditions that require a special diet [36], and more studies are needed to assess this group in Brazil. Additionally, children with other behavioral disorders, such as Autism Spectrum Disorder (ASD), are also prone to FN, with a high prevalence of food neophobia reported in Brazil (73.9%) [37]. In our study, ASD children represent about 20% of the sample, and we included these children as part of a representative sample from the Federal District, given that all aspects related to food refusal can be exacerbated in ASD. This is due to their rigidity, difficulties in social environments, repetitive behaviors, and reluctance to accept new foods or changes in routine [39,40,41]. Furthermore, food refusal in these individuals may be influenced by food group type, smell, texture, color, temperature, and preferences for specific brands and packaging [41].

Mothers were the caregivers who mainly participated, probably because females are more interested in health and childcare [42]. Similar response rates were found in another study in Brazil [19]. This could lead to a sex bias, but it is minimized once the reproducibility of the instrument was assessed between different caregivers (two caregivers responded to the BCFNeo for the same children), and the result was good (ICC > 0.6) [19].

The pandemic may have influenced the results. A meta-analysis showed that one in five children <18y/o are globally experiencing anxiety symptoms, and one in four are globally experiencing depression symptoms at higher levels. These pooled estimates, which increased over time, double the prepandemic estimates [43]. This behavior and other factors caused by the pandemic could reflect changes in food consumption [28]. A systematic review conducted to summarize an overview of changes in eating habits due to the COVID-19 pandemic demonstrated that eating changed qualitatively [44]. The study included 157.900 children and adolescents from 39 full-text articles. The studies were conducted on five continents, and controversial results about eating habits were reported. Despite the results, the authors concluded that consuming fruits and vegetables increased during the COVID-19 pandemic [44]. However, our results showed high FN for fruits, not following the study that showed an increase in fruit intake during the pandemic.

As we had hypothesized, FN does not decrease with age, and there is a difference in FN according to gender. Older children were more neophobic than younger children. Boys are more neophobic than girls. More studies are needed to make local comparisons on FN in Brazilian children. Only two local studies outside the FD were found [25,26]; however, they did not use validated instruments specific to the target audience. It is necessary to compare FN among Brazilian states and to assess the peak of FN in this population since the national study [19] did not see differences in the expression of neophobia concerning age, and ours showed that older children could be more neophobic to some group foods.

Multilevel strategies enable the child to accept new foods and provide food familiarization, social learning, and associative learning [5]. It is necessary to have a holistic view of FN since this behavior is often underestimated and is considered a transitory condition [5]. Understanding the factors involved in FN is vital to making any progress to face it [9]. Effective prevention and treatment strategies depend on the knowledge of researchers and clinicians based on antecedent courses and interactions of FN [5]. Children with FN should be treated by a gastroenterologist, feeding therapist, clinical dietitian, sensory integration therapist, neurologist, psychologist, and other professionals [6]. One of the strengths of this study is that it used a validated instrument for the country, language, and target population, and an appropriate methodology that measures the level of FN through a score. Our result can improve public policies on healthy eating, government actions, and local and national movements and aimed at society. Furthermore, the instrument is divided into domains with their respective scores and questions about the context and environment in which FN occurs. This possibility allows, at the individual and clinical level, to assess which points are most urgent to be addressed and, thus, to evaluate periodically the best strategy to overcome FN, whether it is focused on food groups such as fruits, vegetables, school meals, or environments such as friends’ houses, school, or one’s own home.

## 5. Conclusions

The results allowed us to realize that the children of the FD are more neophobic than Brazilian children from another more extensive study, and between sex, boys are more neophobic. FN does not decrease with age. On the contrary, it can increase, suggesting that the FN needs further study in Brazil and more specific groups. Therefore, it is not easy to know whether increasing age, in this study, was a determinant of the increase in FN. It would be necessary to carry out a longitudinal study to verify this relationship.

Assessing FN with a validated instrument for the target audience enables differentiating the levels of this behavior. Future studies should adopt similar methodologies to ensure the comparability of results and analyze the differences among regions or countries. Our study not only evaluates the degree of FN but also identifies whether there is neophobia toward fruits, vegetables, or food in general. Other research could enhance this diagnosis through observational studies that track children’s behavior during meals. Longitudinal studies may also be useful for examining changes in neophobia over time.

Based on the study data, schools seem to be a promising environment for addressing food neophobia in children. Therefore, it is crucial to implement public policies that encourage both public and private schools to engage in year-round nutrition-focused initiatives, such as cooking workshops, school gardens, and food tastings. These activities can increase children’s exposure to new and regional foods, potentially reducing food neophobia over time.

## Figures and Tables

**Table 1:** Children’s profiles (n = 595) and their sociodemographic data. Federal District/Brazil, 2020–2022.

	Categories	Sample	
		n	%
Sex	Male	336	56.5%
	Female	259	43.5%
Age	4 years old	120	20.2%
	5 years old	93	15.6%
	6 years old	71	11.9%
	7 years old	53	8.9%
	8 years old	90	15.1%
	9 years old	60	10.1%
	10 years old	68	11.4%
	11 years old	40	6.7%
Diagnoses ^a^	No disease	367	61.68%
	Food allergies	31	5.21%
	Food intolerance	36	6.05%
	Autism spectrum disorder	114	19.15%
	Down’s syndrome	29	4.87%
	Eating disorders ^b^	3	0.50%
	Attention deficit hyperactivity Disorder/Anxiety	10	1.68%
	Others diagnoses ^c^	45	7.56%

^a^ Children may have one or more diagnoses. ^b^ Anorexia/bulimia/pediatric eating disorder, ^c^ such as other syndromes, respiratory diseases, and others.

**Table 2:** Neophobia classification (n = 595) and distribution. Federal District/Brazil, 2020–2022.

	Food Neophobia		
	Low (n; %)	Moderate (n; %)	High (n; %)
Domain of food neophobia in general * (FNgen)	126 (21.2%)	185 (31.1%)	284 (47.7%)
Domain of food neophobia for fruits * (FNfru)	188 (31.6%)	187 (31.4%)	220 (37.0%)
Domain of food neophobia for vegetables * (FNveg)	144 (24.2%)	162 (27.2%)	289 (48.6%)
TOTAL INSTRUMENT SCORE ** (BCFNeoTot)	146 (24.5%)	194 (32.6%)	255 (42.9%)

* Domain score: low (up to 13 points); moderate (14 to 21 points); high (22 points or more). ** Total score cutoff points: low—up to 40 points; moderate—from 41 to 65 points; high—66 points or more.

**Table 3:** Food neophobia score and classification distribution by sex and age group (n = 595). Federal District/Brazil, 2020–2022.

	Sex			Age		
	Girls (n = 259)	Boys (n = 336)	*p*	4–7 y (n = 337)	8–11 y (n = 258)	*p*
General neophobia (FNgen)						
Score						
Mean (SD)	19.58 (8.50)	21.25 (8.39)	0.017 *	19.92 (8.63)	21.31 (8.20)	0.047 *
Distribution						
Low (≤13)	58 (22.4%)	68 (20.2%)		82 (24.3%)	44 (17.1%)	
Moderate (14 to 21)	96 (37.1%)	89 (26.5%)	0.011 **	102 (30.3%)	83 (32.2%)	0.068 **
High (≥22)	105 (40.5%)	179 (53.3%)		153 (45.4%)	131 (50.8%)	
Fruit neophobia (FNfru)						
Score						
Mean (SD)	16.47 (8.32)	18.26 (8.39)	0.010 *	17.10 (8.66)	17.98 (8.03)	0.208 *
Distribution						
Low (≤13)	93 (35.9%)	95 (28.3%)		116 (34.4%)	72 (27.9%)	
Moderate (14 to 21)	87 (33.6%)	100 (29.8%)	0.005 **	99 (29.4%)	88 (34.1%)	0.237 **
High (≥22)	79 (30.5%)	141 (42.0%)		122 (36.2%)	98 (38.0%)	
Vegetable Neophobia (FNveg)						
Score						
Mean (SD)	18.91 (8.30)	20.62 (8.34)	0.013 *	19.25 (8.66)	20.69 (7.89)	0.038 *
Distribution						
Low (≤13)	79 (30.5%)	65 (19.3%)		89 (26.4%)	55 (21.3%)	
Moderate (14 to 21)	71 (27.4%)	91 (27.1%)	0.001 *	96 (28.5%)	66 (25.6%)	0.049 **
High (≥22)	109 (42.1%)	180 (53.6%)		152 (45.1%)	137 (53.1%)	
TOTAL (BCFNeoTot)						
Score						
Mean (SD)	54.96 (23.90)	60.13 (23.40)	0.008 *	56.27 (24.70)	59.98 (22.29)	0.056 *
Distribution						
Low (up to 40)	71 (27.4%)	75 (22.3%)		98 (29.1%)	48 (18.6%)	
Moderate (41 to 65)	91 (35.1%)	103 (30.7%)	0.023 **	98 (29.1%)	96 (37.2%)	0.082 **
High (66 or more)	97 (37.5%)	158 (47.0%)		141 (41.8%)	114 (44.2%)	

* Independent *t*-test; ** Mann–Whitney test.

**Table 4:** Score and distribution of food neophobia according to the environment by age group and sex (n = 595). Federal District/Brazil, 2020–2022.

		School Mean (SD)	Home Mean (SD)	Friend’s House Mean (SD)	*p* *
Environment in Fruit Neophobia ^1^	Total (n = 595)	1.96 (1.20) ^A^	2.05 (1.23) ^B^	2.07 (1.17) ^B^	0.000
	Girls (n = 259)	1.80 (1.23) ^A^	1.93 (1.22) ^B^	1.90 (1.21) ^AB^	0.008
	Boys (n = 336)	2.08 (1.16) ^A^	2.14 (1.23) ^AB^	2.20 (1.12) ^B^	0.005
	4–7 y (n = 337)	1.82 (1.22) ^A^	2.02 (1.25) ^B^	2.01 (1.18) ^A^	0.000
	8–11 y (n = 258)	2.14 (1.15) ^A^	2.08 (1.19) ^A^	2.15 (1.15) ^A^	0.829
Environment in Vegetable Neophobia ^2^	Total (n = 595)	2.30 (1.16) ^A^	2.31 (1.23) ^A^	2.42 (1.13) ^B^	0.000
	Girls (n = 259)	2.16 (1.20) ^A^	2.17 (1.21) ^A^	2.29 (1.15) ^A^	0.084
	Boys (n = 336)	2.40 (1.13) ^A^	2.41 (1.24) ^B^	2.53 (1.10) ^B^	0.001
	4–7 y (n = 337)	2.13 (1.20) ^A^	2.24 (1.25) ^B^	2.30 (1.15) ^B^	0.000
	8–11 y (n = 258)	2.51 (1.08) ^A^	2.39 (1.20) ^A^	2.58 (1.08) ^A^	0.117
Neophobia with friends’ influence ^3^	Total (n = 595)	2.16 (1.07) ^A^	2.17 (1.07) ^A^	2.20 (1.06) ^A^	0.248
	Girls (n = 259)	2.01 (1.12) ^A^	2.05 (1.05) ^A^	2.07 (1.07) ^A^	0.380
	Boys (n = 336)	2.28 (1.01) ^A^	2.27 (1.08) ^A^	2.30 (1.05) ^A^	0.576
	4–7 y (n = 337)	2.03 (1.08) ^A^	2.13 (1.08) ^B^	2.12 (1.06) ^B^	0.005
	8–11 y (n = 258)	2.34 (1.02) ^A^	2.23 (1.07) ^B^	2.31 (1.06) ^AB^	0.031

^1^ At school, at home, or friend´s house: the taste of a new fruit; ^2^ at school, at home or friend´s house: the taste of a new vegetable; ^3^ at school, at home or friend´s house: a friend’s acceptance would lead the child to taste the food. * Friedman test followed by Wilcoxon test with Bonferroni correction. Groups with different letters differ significantly.

## Data Availability

The original contributions presented in the study are included in the article and Supplementary Materials, further inquiries can be directed to the corresponding authors.

## Supplementary Materials

The following supporting information can be downloaded at: https://www.mdpi.com/article/10.3390/nu16172962/s1, Table S1: Characterization of caregivers and their children (n = 595). Brasília, Brazil, 2020–2022; Table S2: Average score according to age and sex of the children (n = 595). Brasília, Brazil, 2020–2022.

## References

[1] Pliner P., Hobden K. (1992). Development of a scale to measure the trait of food neophobia in humans. Appetite.

[2] Alley T.R. (2018). Conceptualization and measurement of human food neophobia. Food Neophobia.

[3] Pliner P. (1994). Development of Measures of Food Neophobia in Children. Appetite.

[4] Damsbo-Svendsen M., Frøst M.B., Olsen A. (2017). A review of instruments developed to measure food neophobia. Appetite.

[5] Finistrella V., Gianni N., Fintini D., Menghini D., Amendola S., Donini L.M., Manco M. (2024). Neophobia, sensory experience and child’s schemata contribute to food choices. Eat. Weight Disord.-Stud. Anorex. Bulim. Obes..

[6] Bialek-Dratwa A., Szczepanska E., Szymanska D., Grajek M., Krupa-Kotara K., Kowalski O. (2022). Neophobia-A Natural Developmental Stage or Feeding Difficulties for Children?. Nutrients.

[7] Guzek D., Głąbska D., Lange E., Jezewska-Zychowicz M. (2017). A Polish Study on the Influence of Food Neophobia in Children (10–12 Years Old) on the Intake of Vegetables and Fruits. Nutrients.

[8] Firme J.N., De Almeida P.C., Batistela E., Zandonadi R.P., Raposo A., Botelho R.B.A. (2023). Instruments to Evaluate Food Neophobia in Children: An Integrative Review with a Systematic Approach. Nutrients.

[9] Subramaniam A., Muthusamy G. (2024). Food neophobia: Explored and unexplored terrains. Int. J. Econ. Manag. Account..

[10] Kozioł-Kozakowska A., Piórecka B., Schlegel-Zawadzka M. (2018). Prevalence of food neophobia in pre-school children from southern Poland and its association with eating habits, dietary intake and anthropometric parameters: A cross-sectional study. Public Health Nutr..

[11] Helland S.H., Bere E., Bjørnarå H.B., Øverby N.C. (2017). Food neophobia and its association with intake of fish and other selected foods in a Norwegian sample of toddlers: A cross-sectional study. Appetite.

[12] Cooke L., Carnell S., Wardle J. (2006). Food neophobia and mealtime food consumption in 4-5 year old children. Int. J. Behav. Nutr. Phys. Act..

[13] Kutbi H.A., Asiri R.M., Alghamdi M.A., Albassami M.Z., Mosli R.H., Mumena W.A. (2022). Food neophobia and its association with nutrient intake among Saudi children. Food Qual. Prefer..

[14] Di Nucci A., Pilloni S., Scognamiglio U., Rossi L. (2023). Adherence to Mediterranean Diet and Food Neophobia Occurrence in Children: A Study Carried out in Italy. Nutrients.

[15] Stratton L.M., Vella M.N., Sheeshka J., Duncan A.M. (2015). Food neophobia is related to factors associated with functional food consumption in older adults. Food Qual. Prefer..

[16] Torres T.d.O., Gomes D.R., Mattos M.P. (2021). Factors associated with food neophobia in children: Systematic review. Rev. Paul. Pediatr..

[17] Lafraire J., Rioux C., Giboreau A., Picard D. (2016). Food rejections in children: Cognitive and social/environmental factors involved in food neophobia and picky/fussy eating behavior. Appetite.

[18] Cooke L.J., Haworth C.M.A., Wardle J. (2007). Genetic and environmental influences on children’s food neophobia. Am. J. Clin. Nutr..

[19] de Almeida P.C., Vasconcelos I.A.L., Zandonadi R.P., Nakano E.Y., Raposo A., Han H., Araya-Castillo L., Ariza-Montes A., Botelho R.B.A. (2022). Food Neophobia among Brazilian Children: Prevalence and Questionnaire Score Development. Sustainability.

[20] De Carvalho C.A., Fonsêca P.C.D.A., Priore S.E., Franceschini S.D.C.C., De Novaes J.F. (2015). Food consumption and nutritional adequacy in Brazilian children: A systematic review. Rev. Paul. Pediatr..

[21] Instituto Brasileiro de Geografia e Estatística—IBGE (2020). Pesquisa de Orçamentos Familiares 2017–2018: Análise do Consumo Alimentar Pessoal No Brasil.

[22] de Almeida P.C., Rosane B.P., Nakano E.Y., Vasconcelos I.A.L., Zandonadi R.P., Botelho R.B.A. (2020). Instrument to Identify Food Neophobia in Brazilian Children by Their Caregivers. Nutrients.

[23] Codeplan (2022). Companhia de Planejamento do Distrito Federal Pesquisa Distrital por Amostra de Domicílios—PDAD 2021: Distrito Federal.

[24] Cappelleri J.C., Jason Lundy J., Hays R.D. (2014). Overview of Classical Test Theory and Item Response Theory for the Quantitative Assessment of Items in Developing Patient-Reported Outcomes Measures. Clin. Ther..

[25] Silva T.A., Jordani M.T., da Cunha Guimaraes I.G., Alves L., Moreno Braga C.B., Luz S.d.A.B. (2020). Assessment of eating behavior and food neophobia in children and adolescents from uberaba-mg. Rev. Paul. Pediatr..

[26] De Medeiros R.T.P. (2008). Caracterização da Neofobia Alimentar em Crianças de Três a Seis Anos. Master’s Thesis.

[27] Hazley D., Stack M., Walton J., McNulty B.A., Kearney J.M. (2022). Food neophobia across the life course: Pooling data from five national cross-sectional surveys in Ireland. Appetite.

[28] Tan C.C., Holub S.C. (2012). Maternal feeding practices associated with food neophobia. Appetite.

[29] Knaapila A., Tuorila H., Silventoinen K., Keskitalo K., Kallela M., Wessman M., Peltonen L., Cherkas L.F., Spector T., Perola M. (2007). Food neophobia shows heritable variation in humans. Physiol. Behav..

[30] da Silva Oliveira M.N., Wehrmann M.E.S.d.F., Sauer S. (2015). Agricultura Familiar no Distrito Federal: A busca por uma produção sustentável. Sustentabilidade em Debate.

[31] Companhia de planejamento do Distrito Federal—CODEPLAN, Secretaria de Estado do Planejamento, do Orçamento e Gestão (2015). Agricultura Familiar No Distrito Federal: Dimensões e Desafios.

[32] Ministério Da Educação (2019). Conselho Nacional de Educação Parecer CNE/CEB no 7/2019, Aprovado em 4 de Julho de 2019.

[33] Brazilian Law (2013). Lei No 12.796, de 4 de Abril de 2013.

[34] Damsbo-Svendsen M., Frøst M.B., Olsen A. (2017). Development of novel tools to measure food neophobia in children. Appetite.

[35] Pasikowski S. (2024). Snowball Sampling and Its Non-Trivial Nature. Przegląd Badań Eduk. Educ. Stud. Rev..

[36] IBGE—Instituto Brasileiro De Geografia E Estatística Estatísticas IBGE|Cidades@|Distrito Federal|Panorama. https://cidades.ibge.gov.br/brasil/df/panorama.

[37] Emerson R.W. (2021). Convenience Sampling Revisited: Embracing Its Limitations Through Thoughtful Study Design. J. Vis. Impair. Blind..

[38] Nicklaus S., Boggio V., Chabanet C., Issanchou S. (2005). A prospective study of food variety seeking in childhood, adolescence and early adult life. Appetite.

[39] Lázaro C., Pondé M.P. (2018). Narratives of mothers of autism spectrum disorders subjects: Focus on eating behaviour. Matern. Child Nutr..

[40] Monteiro M.A., dos Santos A.A.A., Gomes L.M.M., Rito R.V.V.F. (2020). Autism Spectrum Disorder: A Systematic Review About Nutritional Interventions. Rev. Paul. Pediatr..

[41] Marshall J., Hill R.J., Ziviani J., Dodrill P. (2014). Features of feeding difficulty in children with Autism Spectrum Disorder. Int. J. Speech-Lang. Pathol..

[42] Davidson D.J., Freudenburg W.R. (1996). Gender and environmental risk concerns: A review and analysis of available research. Environ. Behav..

[43] Racine N., McArthur B.A., Cooke J.E., Eirich R., Zhu J., Madigan S. (2021). Global Prevalence of Depressive and Anxiety Symptoms in Children and Adolescents During COVID-19: A Meta-analysis. JAMA Pediatr..

[44] Pourghazi F., Eslami M., Ehsani A., Ejtahed H.S., Qorbani M. (2022). Eating habits of children and adolescents during the COVID-19 era: A systematic review. Front. Nutr..

