# Supplementary material for: Food Neophobia in Children: A Case Study in Federal District/Brazil
Source: Nutrients. 2024 Sep 3;16(17):2962. doi: 10.3390/nu16172962 (PMC11397450; doi:10.3390/nu16172962)
Supplement: Supplementary file 1 [file nutrients-16-02962-s001.zip › nutrients-3159209-supplementary.pdf]

Table S1. Characterization of caregivers and their children (n=595). Brasília, Brazil, 2020-2022.

	Categories	Sample	
		**n**	**%**
**Caregivers Sex**	Male	37	6.2%
	Female	558	93.8%
**Caregivers' degree of kinship**	Mothers	534	89.7%
	Fathers	32	5.5%
	Sisters or brothers	8	1.3%
	Stepmothers	3	0.5%
	Aunts	6	1.0%
	Cousins	4	0.7%
	Grandparents	8	1.3%
**Caregiver Marital status**	Single	57	9.6%
	Married/ stable union	489	82.2%
	Separate/ divorced	43	7.2%
	Widower	6	1.0%
**Educational Level of Caregivers**	From 1st to 4th grade of elementary school	4	0.7%
	From 5th to 8th grade of elementary school	5	0.8%
	High School (incomplete)	11	1.8%
	High school (complete)	48	8.1%
	Undergraduate (incomplete)	47	7.9%
	Undergraduate (complete)	131	22.0%
	Postgraduate	255	42.9%
	Master's degree	68	11.4%
	PhD	20	3.4%
	Postdoctoral	6	1.0%
**Housing area**	Urban area	586	98.5%
	Rural area	9	1.5%
**Number of Residents**	Two	33	5.5%
	Three	159	26.7%
	Four	303	51.0%
	Five	71	12.0%
	Six	21	3.5%
	Seven	5	0.8%
	Eight	2	0.3%
	Ten	1	0.2%
**Monthly family income**	No income	4	0.7%
	Up to 1 MW	31	5.2%
	Up to 2 MW	34	5.7%
	Up to 3 MW	26	4.4%
	Up to 4 MW	27	4.5%
	Up to 5 MW	37	6.2%

	Up to 6 MW	29	4.9%
	Up to 7 MW	18	3.0%
	Up to 8 MW	36	6.1%
	Up to 9 MW	25	4.2%
	From10 and 12 MW	89	15.0%
	From 13 and 15 MW	57	9.6%
	Above 15 MW	133	22.4%
	Not informed	49	8.1%
**Caregivers age**	Up to 20 y/o	9	1.5%
	From 21 and 30 y/o	51	8.6%
	From 31 and 40 y/o	291	48.9%
	From 41 and 50 y/o	219	36.8%
	From 51 and 60 y/o	16	2.7
	From 61 and 70 y/o	9	1.5%

Tabela S2. Average score according to age and sex of the children (n=595). Brasília, Brazil, 2020-2022.

Sex	Age	Mean FNfru	p*	Mean FNveg	p*	Mean FNgen	p*	Mean BCFNeo Tot	p*
1	4 y/o	16.90 ± 8.65	0.002	19.11 ± 8.66	0.000	19.53 ± 8.52	0.001	55.54 ± 24.18	0.000
2	4 y/o	19.49 ± 8.21		22.03 ± 7.93		22.21 ± 7.86		63.63 ± 22.50	
1	5 y/o	17.10 ± 8.51	0.315	20.15 ± 8.47	0.560	20.17 ± 8.89	0.498	57.42 ± 24.46	0.690
2	5 y/o	18.00 ± 8.68		19.64 ± 8.23		20.80 ± 8.83		58.44 ± 24.38	
1	6 y/o	15.73 ± 8.68	0.003	18.40 ± 8.43	0.002	19.00 ± 8.47	0.005	53.12 ± 23.87	0.002
2	6 y/o	18.92 ±9.29		21.44 ± 8.53		21.89 ± 8.95		62.25 ± 25.21	
1	7 y/o	15.97 ± 8.01	0.000	18.96 ± 7.72	0.005	18.35 ± 7.85	0.000	53.27 ± 21.96	0.000
2	7 y/o	20.07 ± 8.01		21.93 ± 7.89		22.42 ± 8.66		64.42 ± 22.87	
1	8 y/o	17.48 ± 8.80	0.275	20.44 ± 7.96	0.291	20.08 ± 9.14	0.147	58.00 ± 23.88	0.191
2	8 y/o	18.72 ± 8.34		21.54 ± 7.79		21.75 ± 8.07		62.00 ± 22.21	
1	9 y/o	16.02 ± 8.43	0.000	18.41 ± 8.17	0.001	18.78 ± 8.59	0.000	53.21 ± 23.41	0.000
2	9 y/o	20.27 ± 8.70		22.04 ± 8.31		22.83 ± 8.53		65.14 ± 24.04	

1	10y/o	16.17 ± 8.36	0.004	18.55 ± 8.06	0.015	18.66 ± 8.94	0.002	53.39 ± 23.96	0.003
2	10 y/o	19.72 ± 8.64		21.49 ± 8.67		22.60 ± 8.50		63.81 ± 23.96	
1	11 y/o	15.56 ± 8.53	0.005	17.79 ± 8.80	0.010	19.07 ± 9.56	0.020	52.42 ± 25.61	0.007
2	11 y/o	19.68 ± 9.02		21.59 ± 8.59		22.82 ± 9.26		64.10 ± 25.36	

1-Female; 2-Male; * Student’s t test
